# Clinical Significance of Claudin Expression in Oral Squamous Cell Carcinoma

**DOI:** 10.3390/ijms231911234

**Published:** 2022-09-23

**Authors:** Tatjana Zejc, Jörg Piontek, Jörg-Dieter Schulzke, Michael Fromm, Jürgen Ervens, Rita Rosenthal

**Affiliations:** 1 Department of Gastroenterology, Rheumatology and Infectious Diseases, Clinical Physiology/Nutritional Medicine, Campus Benjamin Franklin, Charité—Universitätsmedizin Berlin, 12203 Berlin, Germany; 2 Klinik für HNO-Heilkunde, Kopf- und Halschirurgie, Plastische Chirurgie, Vivantes Klinikum Neukölln, Rudower Straße 48, Neukölln, 12351 Berlin, Germany

**Keywords:** oral squamous cell carcinoma, OSCC, oral cancer, prognosis, reduced survival, claudin, tight junction

## Abstract

A change in claudin expression has been demonstrated in various tumors. The present study specifically compares claudin expression in oral squamous cell carcinoma (OSCC) with healthy oral epithelium from the same individual and analyzes the association between claudin expression and the clinically relevant course parameters. Our study includes tissue samples and clinically relevant follow-up data from 60 patients with primary and untreated OSCC. The oral mucosa was analyzed via Western blot for the expression of claudin-1, -2, -3, -4, -5, and -7. Importantly, the tumor and healthy tissues were obtained pairwise from patients, allowing for intraindividual comparisons. Both the healthy and tumor epithelium from the oral cavity did not express the claudin-3 protein. The intraindividual comparison revealed that, in OSCC, claudin-2 expression was higher, and the expression of claudin-4, -5, and -7 was lower than in healthy epithelium. An association was found between increased claudin-2 expression and shorter relapse-free survival. In addition, the reduced expression of claudin-4 had a negative impact on relapse-free survival. Furthermore, associations between the reduced expression of claudin-7 and the stage of a tumor, or the presence of lymph node metastases, were found. Thus, the expression level of claudin-2, -4, and -7 appears to be predictive of the diagnosis and prognosis of OSCC.

## 1. Introduction

Oral squamous cell carcinoma (OSCC) represents approximately 2% of all malignant tumors worldwide. Approximately 355,000 new cases of OSCC were dated worldwide in 2018. A total of 177,400 people died of the disease in the same year. The frequency in the clinical picture of oral cancer shows regional differences. The age-standardized incidence rate of OSCC in Melanesia is comparable to that of colon cancer in Western Europe [1]. It is believed that tumorigenesis can be associated with the de-differentiation of cells with a loss of cell polarity and cell integrity, with accompanying tight junction (TJ) dysfunction. TJs are the most apical cellular structures between adjacent cells, forming a selective paracellular barrier and maintaining cell polarity. Additionally, TJs are involved in the regulation of epithelial functions, cellular differentiation, and intracellular signaling [2,3,4]. The TJ is composed of several integral membrane proteins and associated proteins. One main class of membrane proteins is the claudin family, comprising 27 members in mammals, which are expressed in a tissue-specific pattern [5]. A claudin dysregulation was described in various types of cancer, with some claudins being upregulated in certain types of cancer but being downregulated in others [6,7,8]. It has not been clarified yet whether claudin dysregulation contributes to or is a consequence of tumorigenesis. Claudin-1, -3, -4, and -7 are among the most frequently dysregulated claudins in different tumors [7,9]. In squamous cell carcinoma of the skin, changes in claudin-1, -2, -4, and -7 were described [10,11]. This altered expression of the claudin family members in tumor tissue makes them important as diagnostic and especially prognostic markers.

In OSCC, claudin-1, -2, -3, -4, -5, -7, and -8 have already been investigated; in most of the studies, this has been carried out immunohistochemically [12,13,14,15,16,17], and only in a few studies has it been analyzed at the gene expression level [18,19]. However, the results are inconsistent, and in nearly all studies the comparison with the healthy oral mucosa of the same individual was missing. None of the studies using immunohistochemical methods analyzed claudin-8; it was detected only in one study on the gene expression level [19], and claudin-8 was also excluded from this study. Therefore, the aim of this study was to examine primary OSCC for the expression of the TJ proteins claudin-1 to -5 and claudin-7 and to compare this with expression in healthy oral mucosa tissue of the same patient in order to determine factors relevant to the prognosis. For this purpose, 60 patients with primary untreated OSCC were recruited, and their resident tumors and respective healthy oral controls were analyzed by protein analysis.

## 2. Results

### 2.1. Missing Claudin-3 Expression in OSCC and Healthy Oral Epithelium

All patients were analyzed concerning the expression of claudin-3 in tumor and control tissues since an immunohistochemical study presented claudin-3 in OSCC and in the healthy oral epithelium [20]. Western blot analysis did not detect a band at the expected molecular weight of about 23 kDa, but did reveal a band at about 55 kDa in all patients (see Supplementary Materials, Figure S1), which could possibly represent a multimer of claudin-3 or a complex of claudin-3 with another protein. Further analyses using a pulldown assay and mass spectroscopy did not detect claudin-3 in healthy cheek mucosa. We, therefore, define this band at 55 kDa as non-specific, and conclude that the claudin-3 protein is not expressed in healthy cheek mucosa and OSCC.

### 2.2. Claudin Expression in OSCC

The present study compares the expression of claudin-1, -2, -4, -5, and -7 in OSCC and in the healthy tissue of the same individual in 60 patients with tumor localization in different parts of the oral cavity. Table 1 summarizes the number of patients with unchanged, increased, or decreased expression of the investigated claudins.

Overall, there was no significant difference in claudin expression change in OSCC in different areas of the oral cavity (see Supplementary Materials, Figure S2). Therefore, the results of claudin expression change in all investigated patients were considered for all analyses regardless of the location within the oral cavity.

**Figure 1:**
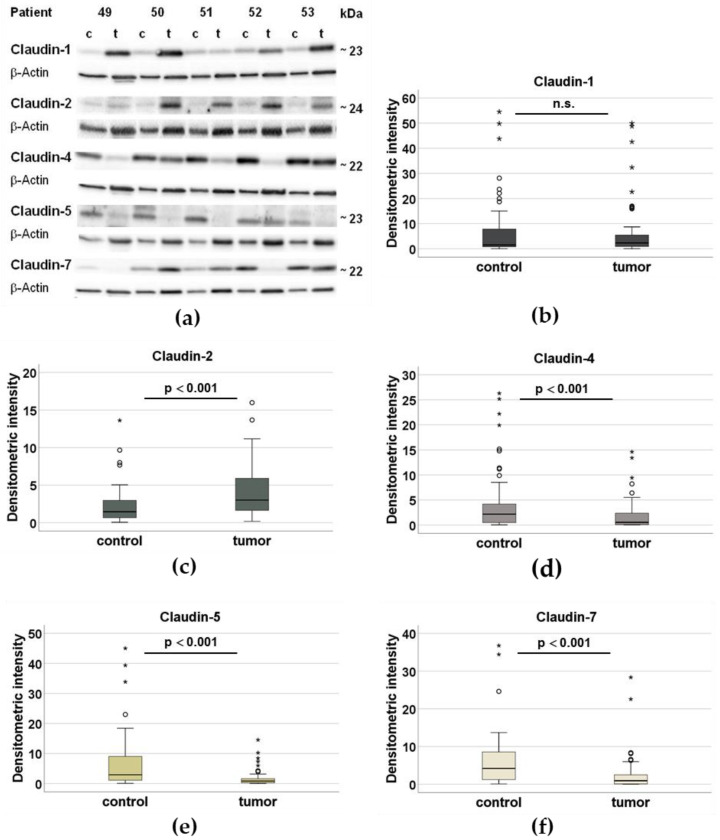
Analysis of claudin expression using Western blots. (**a**) Western blot analysis of the claudins in the control tissue (c) and the respective tumor tissue (t) of patients 49–53, normalized by the loading control ß-actin (42 kDa). The intra-individual comparison between the tumor and the control is carried out on the basis of the intensity of the bands. (**b**–**f**) In box plots, densitometric analyses of the expression of claudin-1, claudin-2, claudin-4, claudin-5, and claudin-7 in the healthy control tissue of the 60 patients (n = 60) and the tumor tissue (n = 60), normalized and represented with the loading control β-actin (° outlier, * extremal value, significantly different from control *p* < 0.001, n.s. not significant).

In OSCC, the expression of claudin-4, -5, and -7 was lower compared to the oral mucosa of the intraindividual control (*p* < 0.001). In contrast, claudin-2 was more strongly expressed in OSCC (*p* < 0.001). The expression level of claudin-1 showed no significant difference between the tumor and the control tissue (*p* = 0.596). Claudin-1 was upregulated in the OSCC in some patients, whereas it remained unchanged or downregulated in other patients. The differences in protein expression for all the investigated claudins are illustrated in Figure 1. In addition, the complete Western blot analysis for 60 tumors and the respective controls is shown in Supplementary Materials, Figure S3.

### 2.3. Claudin Expression and Clinicopathological Parameters

An association between the expression of claudin-1, -2, -4, -5, and -7 in the tumor and control tissues and the clinicopathological parameters (UICC stage, tumor expansion, lymph node status, histological differentiation, lymphovascular invasion, age of manifestation, smoking and alcohol consumption, and claudin-dependent recurrence-free survival) was checked. The staging was performed according to UICC 2009 (7th edition) since the patients were recruited from April 2014 to December 2016. A staging, according to the current UICC (8th edition, 2017), could not be performed since the T-stage could not be reclassified due to missing data on the invasion depth. A reclassification of the lymph node status, according to the current edition, revealed no differences to the 7th edition concerning the status and differences in claudin expression between the tumor and control tissues (Supplementary Materials, Tables S1 and S2).

No relevant connection between clinical pathological parameters and claudin-1, -2, -4, and -5 could be demonstrated. In contrast, the reduced expression of claudin-7 in oral OSCC significantly correlates with lymph node metastasis (*p* = 0.004) and an advanced UICC stage (*p* = 0.008) (see Table 2).

### 2.4. Claudin Expression Changes and Recurrence-Free Survival

**Figure 2:**
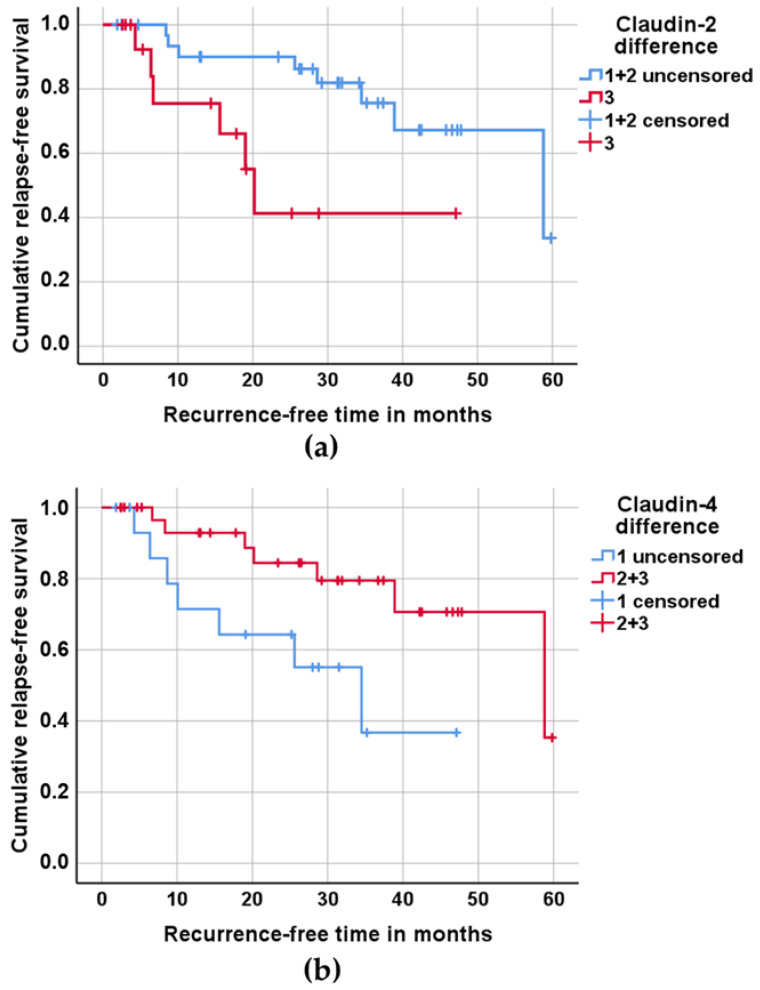
Recurrence-free survival of 49 patients treated and followed up by curative treatment, depending on claudin expression. (**a**) For this analysis, the patients were divided into three groups of equal size based on the differences in claudin-2 expression between tumor tissue and healthy control tissue; group 1, with nearly as much claudin-2 in the tumor tissue as in the control tissue; group 3, with the highest difference in claudin-2 expression between the tumor tissue and control tissue. Groups 1 and 2 were merged for pairwise comparison between groups 1 + 2 and 3 (*p* = 0.01). (**b**) For this analysis, the patients were divided into three groups of equal size based on the differences in claudin-4 expression between the tumor tissue and healthy control tissue, with the largest difference between tumor and control in group 1 and the smallest or no difference in group 3. Groups 2 and 3 were merged for pairwise comparison between group 1 and groups 2 + 3 (*p* = 0.029). P-values of log-rank analysis.

The Kaplan–Meier method was used for the association between claudin expression changes and recurrence-free survival. The analysis was based on the data of 49 patients who were treated curatively and followed up. For the use of this method, the patients were divided into three groups of equal size dependent on changes in claudin expression between the tumor and control tissues (see Supplementary Materials, Table S3). For claudin-2, which is upregulated in OSCC, the tumor tissue from group 1 had nearly as much claudin-2 as the control tissue; groups 2 and 3 showed a higher expression of claudin-2 in the tumor tissue. For claudin-4, which is downregulated in OSCC, group 1 exhibited the largest difference between the tumor and control tissues, and group 3 saw the smallest or no difference. For the Kaplan–Meier survival analysis (see Figure 2), groups 1 and 2 for claudin-2 and groups 2 and 3 for claudin-4 were merged, as there was no significant difference in the recurrence-free time. The Kaplan–Meier survival analysis revealed that an increased claudin-2 expression is associated with a shorter recurrence-free time (see Figure 2a), whereas the decreased expression of claudin-4 shows an association with a shortened recurrence-free time (see Figure 2b).

### 2.5. Claudin-2 Expression as an Independent Significant Prognostic Factor

Further calculations using a Cox multi-regression analysis examined the influence of several co-variables (claudin expression difference, age, tumor stage, lymph node status, and histological differentiation) for recurrence-free survival. These analyses revealed that, exclusively, the increase in claudin-2 expression is an independent significant prognostic factor for recurrence-free survival in addition to the tumor stage and lymph node status (see Table 2). A classification of the lymph node status, according to UICC 2017, was possible in 36 cases; the Cox multi-regression analysis revealed similar results, as shown in Table 3 (see Supplementary Materials, Tables S4 and S5). In addition, we performed a ROC analysis for differences in claudin-2 and claudin-4 expression and recurrence within two years. This analysis revealed the influence of claudin-2 expression (*p* = 0.025), whereas claudin-4 expression has no significant effect.

## 3. Discussion

In the present study, claudin expression was examined in OSCC, for which only a few studies are available. This is the first study that performed an intraindividual quantitative comparison between OSCC and non-affected oral mucosa using Western blot analysis. The study analyzed samples from oral mucosa (of the cheek) and OSCC from 60 patients and showed that claudin expression in OSCC had changed compared to the healthy oral mucosa from the same individual and that these changes are partially associated with clinicopathological features.

The tumor localization within the oral cavity had no significant influence on the level of claudin expression in the present work (see Supplementary Materials, Figure S2). The same results were found in the studies of Lourenco et al. [20], who investigated the same claudins as we did, and of Babkair et al., who analyzed claudin-1 [21]. In contrast, the study by Ouban et al. reported higher claudin-1 expression in tongue carcinoma compared to other oral OSCC, although this was not statistically significant [22].

Claudin-1 expression in OSCC did not differ from that of the intraindividual control tissue, and there was no significant association between claudin-1 expression and clinicopathological features. In contrast to our results, other studies, which assessed claudin-1 semi-quantitatively via immunohistochemistry, reported an increase in claudin-1 expression in OSCC without any effect on overall survival [16] in connection with an advanced tumor stage [23], the poor [23,24] or good differentiation of oral tumor tissue [20,22,25], lymph node metastasis, and angiolymphatic and perineural invasion [23]. Most of the studies have a commonality in that, in contrast to our study, they did not examine the healthy oral control tissue from the same individual. It is obvious (see Supplementary Materials Figure S3) that people do not have the same amount of claudins in their normal, as well as neoplastic-modified, epithelium in the oral cavity, even when comparing age- and sex-matched patients. No other study concerning oral squamous epithelium and its benign or malignant changes has performed a quantitative analysis of this.

A comparison of claudin-1 expression and localization in squamous cell carcinoma (SCC) and healthy skin tissue was performed by immunostaining analysis. One study revealed the strong expression of claudin-1 in well-differentiated areas of cutaneous SCC [26], whereas another study reported a reduction in claudin-1 in skin SCC [11]. Additionally, changes in claudin-2, -4, and -7 and other TJ proteins, such as ZO-1 and occludin, were found in SCC of the skin [11,26], but the association between claudin expression and tumor characteristics has not been analyzed so far.

In the present study, an increase in claudin-2 expression in OSCC was found at the protein level compared to the intraindividual oral control. Although an increase in claudin-2 expression was found, claudin-2 expression is ubiquitously present in normal and neoplastic tissue, so the benefit of potential therapy against claudin-2 (as in the case of claudin-18.2 in advanced gastric carcinoma [27]) appears to be restricted. However, we identified claudin-2 as an independent variable for relapse-free survival. Patients with an increase in claudin-2 expression have shown shorter relapse-free survival compared to patients with approximately normal claudin-2 expression. Patients with an increase in claudin-2 expression lived for an average of 12 months without recurrence, in contrast to patients with approximately the same expression of claudin-2 in healthy and in tumor tissue of the oral cavity, who were free of recurrence, on average, for 34 months. This result is in contrast to another study using semi-quantitative analysis to measure claudin-2 expression in head and neck squamous cell carcinoma [16], which showed that lower claudin-2 levels were associated with poorer outcomes. A similar result regarding increases in the expression of claudin-2 in primary breast cancer and reduced relapse-free survival was observed [28]. An upregulation of claudin-2 was also detected in cutaneous SCC [11], colorectal cancer [29,30], and endometrioid endometrial adenocarcinoma [31], and in two-thirds of examined lung adenocarcinoma samples [32]. It has been shown that altered claudin-2 expression influences epithelial barrier function, and also modulates various cellular processes like proliferation, migration, cell survival, and epithelial-mesenchymal transition [33]. All these effects might contribute to tumor growth and invasion and thereby shorten relapse-free survival in patients with increased claudin-2 expression of OSCC.

We noticed that claudin-3 is not expressed in the healthy cheek mucosa and the OSCC. This result is in contrast to the only published study that was able to detect immunohistochemically claudin-3 in almost 70% of OSCC and in the healthy oral epithelium [20]. In the present study, we detected a band at approximately 55 kDa in each Western blot experiment. This band does not represent a multimer of claudin-3 or a claudin-3-protein complex. This finding may indicate that the claudin-3 antibody tends to bind non-specifically. In fact, the study by Castro Dias et al. reported the non-specificity of claudin-3 antibodies; they detected a claudin-3 signal in Western blot analysis and in immunofluorescence staining in claudin-3-deficient mice [34]. This could explain the detection of anti-claudin-3 immunoreactivity in the healthy oral epithelium and the OSCC in the study of Lourenco et al. [20].

We were able to demonstrate that claudin-4 expression is lower in OSCC than in the healthy oral epithelium. This supports the immunohistochemical study of Sappayatosok et al., which showed that claudin-4 was detected in 60% of the examined tumors in less than 25% of the cells [23]. The patients in the present study, who had approximately the same expression of claudin-4 in the healthy cheek mucosa and in the OSCC, lived without recurrence for almost twice as long as those who had reduced claudin-4 expression. The result of the present study correlates with the immunohistochemical study of Vicente et al., which found no recurrence of OSCC in patients with strong claudin-4 expression [35]. For tumors in other locations, such as esophageal carcinoma and hepatocellular carcinoma, a reduced claudin-4 expression was also associated with shortened recurrence-free survival [36,37].

We could not establish any association between claudin-5 expression and clinicopathological factors. The comparably weak expression of claudin-5 in immunohistochemical stains [12], or even the absence of claudin-5 in over 50% [20] to 75% [38] of OSCC cases, agrees with the reduced expression of claudin-5 in the intraindividual comparison between the healthy and neoplastic-modified oral epithelia in the Western blot analysis of the present study.

In this intraindividual comparison, an association between the reduced expression of claudin-7 and an advanced UICC stage could be determined. In addition, the reduced expression of claudin-7 in OSCC correlates with the presence of lymph node metastases and can thus act as a predictive marker for positive lymph node involvement. This is in line with other studies [14,20,38,39]. A recent study on an OSCC cell line presented that the overexpression of claudin-7 inhibits the proliferation, invasion, and migration of OSCC cells [40]. In the future, the reduction of claudin-7 expression in OSCC (compared to control oral tissue) can serve as a basis for decisions that are for or against lymph node dissection.

We have to mention that the survival rate in this study was relatively high when compared to the five-year survival rate of patients with this cancer type, which is lower than 50% [41]. Here, only four patients died during the observation time. This may be due to the relatively short observation time with a mean value of 21 ± 17 months (mean ± SD), ranging from 0.1 months (no further observation after the surgery) to 59.8 months.

In summary, the expression of claudin-2, claudin-4, and claudin-7 changed in OSCC compared to the healthy oral tissue in the same patient, and these expression changes are associated with clinicopathological features.

## 4. Materials and Methods

### 4.1. Patient and Sample Collection

From April 2014 to December 2016, the samples of 63 patients were taken and investigated, 60 patients with histologically confirmed and untreated OSCC of the oral cavity were enrolled in this study. After histological confirmation and approval from the institutional tumor board, all patients underwent surgery according to the S3 guidelines for oral cancers, including neck dissection, tumor resection, and primary reconstruction if necessary at Charité–Universitätsmedizin Berlin, Campus Benjamin Franklin. Samples of the OSCC and oral mucosa from the cheek of the non-tumor side of the patient were taken before tumor resection and immediately frozen in liquid nitrogen and stored at −80 °C.

The information on patient data and the tumor stage were taken from the patient data system of the clinic. Before enrolling in the study, the patients were informed and gave their written consent to participate in the study. The ethics application (EA4/152/15) for this study has been approved by the Charité Ethics Committee.

### 4.2. Patient Characteristics

Patients were pseudonymized by assigned numbers, 1 to 63 (see Supplementary Materials, Figures S1 and S3); patients 19, 49, and 55, with oropharyngeal carcinomas, were excluded from this study. The patient group includes both men and women, and every tumor stage according to UICC 2009 was represented at least once. A staging, according to the current UICC (8th edition, 2017), could not be performed, since the T-stage could not be reclassified due to missing information on tumor depth. Most tumors (n = 38) were moderately differentiated (G2-stage). The mean observation time taken from the hospital’s patient data acquisition system was 21.0 months, with a minimum of 0.1 months and a maximum of 59.8 months. The patient characteristics are summarized in Table 4.

### 4.3. Western Blot Analysis

For Western blot analysis, total protein extraction from the OSCC resectate and the healthy oral squamous epithelium of the opposite cheek was carried out with a highly denaturing RIPA (Radioimmunoprecipitation assay) lysis buffer (25 mM HEPES, 2 mM EDTA, 25 mM NaF, 1% SDS), pH adjusted to 7.6, with 1 complete mini EDTA-free protease inhibitor tablet (Roche)/10 mL buffer). Small lentil-sized samples of healthy and malignant tissues were homogenized in 1 mL of the lysis buffer, incubated on ice for 30 min, sonicated in a pulsatile manner, and passed through 26-G 3/8-in needles. The protein content of the lysates was determined using bicinchoninic acid (BCA) protein assay reagent (Pierce, Rockford, IL, USA) and quantified with a plate reader (Tecan, Zürich, Switzerland). The protein content of the lysates was adapted, and the SDS-PAGE (sodium dodecyl sulfate polyarylamide gel electrophoresis) was then carried out according to Laemmli, with 12.5% acrylamide separating gel and 20 µg protein in a 20 µL sample in each gel bag. After the proteins were sorted according to their size in the SDS-PAGE, the proteins were transferred to a PVDF membrane (Perkin Elmer, Boston, MA, USA) during the Western blot using a vertical electric field. The transfer was carried out using the wet Western blot process.

For immunodetection of the proteins, with specific antibodies (see Table 5), the primary antibody, diluted 1:1000 in TBST (Tris-buffered saline with Tween20) washing solution with 5% BSA (Bovine Serum Albumin Factor V, Biomol GmbH), was added to the membrane, which was incubated overnight at 4 °C.

After three washing steps, the membrane was incubated with the secondary antibody, diluted 1:10000 in TBST washing solution with 1% blocking reagent (milk powder) for one hour at room temperature. The signal of the secondary antibody coupled to peroxidase with luminol from Lumilight Western Blotting Substrate (Roche Diagnostics GmbH) was detected in Fusion × 7 chemiluminescence and fluorescence imaging (Vilber Lourmat, France). The intensity of the chemiluminescence enabled the quantitative analysis of the amount of the protein to be examined, which was subsequently analyzed densitometrically with AIDA (Advanced Image Data Analyzer Version 3.21, Raytest, Straubenhardt, Germany). The expression strength of the control protein ß-actin was included in the quantitative analysis.

### 4.4. Statistical Analysis

The data were analyzed using the IBM SPSS Statistics, version 25.0 (IBM Corp., Armonk, NY, USA). The difference between claudin expression in the tumor and in the control was checked for significance using the Wilcoxon test and adjusted for multiple testing using the Bonferroni method. The influence of localization (within the oral cavity) on claudin expression was checked using the Kruskal–Wallis test. The associations of claudin-1, -2, -4, -5, and -7-expression with the clinicopathological properties of the tumor were analyzed either with the Spearman–Rho test or with the Mann–Whitney-U test. The relapse-free survival based on claudin expression was estimated using the Kaplan–Meier method and checked for significance using the log-rank test. The recurrence-free survival time is defined by the interval between resection of the primary tumor and the last follow-up in which the patient was in remission (censored, patient without second tumor) or the date of the first occurrence of the recurrence (uncensored, patient with second tumor). In addition, a Cox multivariate regression analysis was carried out to check the influence of several variables on recurrence-free survival. The Kaplan–Meier analysis and the Cox multi-regression analysis was performed with the data from 49 patients, who were treated curatively and followed up. A *p*-value of less than 0.05 (two-tailed) in the tests was considered significant.

## 5. Conclusions

Claudin-2, together with claudin-4, can act as a predictive marker for recurrence-free survival in OSCC. It would therefore be advisable to re-evaluate the patients with an increased expression of claudin-2 and a reduced expression of claudin-4 at shorter intervals in the follow-up assessment of the tumor. In addition, the association between reduced expression of claudin-7 in OSCC and the presence of lymph node metastases should be considered for decisions concerning lymph node dissection. In summary, analyzing the expression of claudin-2, claudin-4, and claudin-7 in OSCC and healthy oral tissue could have importance for everyday clinical practice and improve the quality of diagnosis, prognosis, and therapy for patients with OSCC.

## Figures and Tables

**Table 1:** Number of patients with unchanged, decreased, or increased claudin expression in the tumor tissue compared to the corresponding healthy oral tissue. A claudin signal in the tumor tissue of between 95 and 105% in the healthy tissue is defined as unchanged.

Claudin Expression Compared to Healthy Tissue	Unchanged	Increased	Decreased
Claudin-1	4	27	29
Claudin-2	2	43	15
Claudin-4	0	4	56
Claudin-5	0	3	57
Claudin-7	2	7	51

**Table 2:** Claudin expression in the OSCC and clinicopathological features.

Clinicopathological Features	Number	Claudin-1	Claudin-2	Claudin-4	Claudin-5	Claudin-7
Age at diagnosis	60	r = −0.092 *p* = 0.484	r = 0.072 *p* = 0.583	r = 0.159 *p* = 0.225	r = −0.0612 *p* = 0.641	r = −0.028 *p* = 0.831
Sex: Male Female	38 22	R = 33.08 R = 26.05 *p* = 0.133	R = 29.12 R = 32.86 *p* = 0.425	R = 28.61 R = 33.77 *p* = 0.269	R = 31.45 R = 28.86 *p* = 0.581	R = 28.63 R = 33.73 *p* = 0.276
Nicotine: yes no Not specified	34 7 19	R = 22.15 R = 15.43 *p* = 0.177	R = 21.56 R = 18.29 *p* = 0.510	R = 21.15 R = 20.29 *p* = 0.862	R = 20.74 R = 22.29 *p* = 0.755	R = 21.38 R = 19.14 *p* = 0.652
Alcohol: yes no Not specified	26 2 32	R = 14.85 R = 10 *p* = 0.422	R = 14.15 R = 19 *p* = 0.422	R = 13.88 R = 22.50 *p* = 0.153	R = 14.31 R = 17 *p* = 0.656	R = 14.04 R = 20.50 *p* = 0.284
UICC stage (0–IVa,b,c) Not specified	52 8	r = 0.160 *p* = 0428	r = 0.090 *p* = 0.965	r = 0.005 *p* = 0.560	r = 0.085 *p* = 0.208	r = −0.367 *p* = 0.008 **
T stage (Tis–T4) Not specified	59 1	r = −0.089 *p* = 0.503	r = 0.042 *p* = 0.752	r = −0.227 *p* = 0.083	r = −0.209 *p* = 0.112	r = −0.213 *p* = 0.106
Lymph node status (N0–N2a,b,c) Not specified	54 6	r = 0.150 *p* = 0.279	r = −0.018 *p* = 0.898	r = −0.014 *p* = 0.920	r = 0.010 *p* = 0.940	r = −0.387 *p* = 0.004 **
Histological differ entiation (G1–G3) Not specified	52 8	r = 0.040 *p* = 0.780	r = −0.073 *p* = 0.609	r = 0.006 *p* = 0.965	r = 0.047 *p* = 0.740	r = −0.046 *p* = 0.747
Lymphovascular invasion: yes no Not specified	6 41 13	R = 34.17 R = 22.51 *p* = 0.052	R = 21.50 R = 24.37 *p* = 0.633	R = 29.17 R = 23.24 *p* = 0.323	R = 30.67 R = 23.02 *p* = 0.202	R = 25 R = 23.85 *p* = 0.848

Calculation with the Spearman-Rho or Mann-Whitney-U test with a significant result at ** *p* <0.01. (r = rank correlation coefficient; R = middle rank).

**Table 3:** Cox multi-regression analysis.

Clinicopathological Features	B	Significance	Exp(B)	95% Confidence Interval for Exp(B)	
				Lower	Upper
Claudin-2 difference	0.193	0.023 *	1.213	1.027	1.434
Age at diagnosis	0.005	0.893	1.005	0.937	1.077
T stage	−0.920	0.024 *	0.398	0.179	0.887
Lymph node status	1.149	0.019 *	3.154	1.212	8.212
Histological differentiation	−0.463	0.596	0.629	0.114	3.489

Claudin-2 expression difference in the healthy and tumor tissue of 49 patients; T-stage and lymph node status function as independent variables in relapse-free survival. Significant result at * *p* < 0.05. (B—regression coefficient, Exp(B) Odds ratio).

**Table 4:** Patients’ characteristics.

Clinicopathological Features	Number (%)
**Age at diagnosis**	35–90 years (Ø 65.7)
**Site**	
Tongue	16 (27%)
Floor of mouth	15 (25%)
Alveolar ridge upper and lower jaw	14 (23%)
Multifocal (2 or more localizations)	10 (17%)
Cheek mucosa	3 (5%)
Palate	2 (3%)
**Sex**	
Male	38 (63.3%)
Female	22 (36.7%)
**Nicotine**	
yes	34 (56.7%)
no	7 (11.7%)
not specified	19 (31.7%)
**Alcohol**	
yes	26 (43.3%)
no	2 (3.3%)
not specified	32 (53.3%)
**UICC stage**	
0	1
I	8
II	12
III	11
IV a, b, c	20
not specified	8
**T stage**	
Tis	1 (1.7%)
T1	16 (26.7%)
T2	21 (35%)
T3	6 (10%)
T4	15 (25%)
not specified	1 (1.7%)
**Lymph node status**	
N0	27 (45%)
N1	10 (16.7%)
N2 a, b, c	17 (28.3%)
not specified	6 (10%)
**Histological differentiation**	
G1	4 (7%)
G2	38 (63%)
G3	10 (17%)
not specified	8 (13%)
**Lymphovascular invasion**	
yes	6 (10%)
no	41 (68.3%)
not specified	13 (21.7%)
**Recurrence**	
yes	14 (23.3%)
no	35 (58.3%)
lost follow-up	7 (11.7%)
died	1 (1.7%)
progression in palliative setting	3 (5%)

**Table 5:** Primary and secondary antibodies.

Antibody	Origin	Target	Concentration	Company
Primary	rabbit	claudin-1, -2, -3, -5, -7	1:1000	Thermo Fisher Scientific, Waltham MA, USA
	mouse	claudin-4	1:5000	
	mouse	claudin-5	1:1000	
	mouse	β-actin	1:10,000	Sigma-Aldrich Chemie, Steinheim, Germany
Secondary	goat	rabbit/mouse IgG-F(ab′)_2_ fragment (HRP)	1:10,000	Jackson Immuno Res., West Grove PA, USA

## Data Availability

Not applicable.

## Supplementary Materials

The supporting information can be downloaded at: https://www.mdpi.com/article/10.3390/ijms231911234/s1.

## References

[1] Bray F., Ferlay J., Soerjomataram I., Siegel R.L., Torre L.A., Jemal A. (2018). Global cancer statistics 2018: GLOBOCAN estimates of incidence and mortality worldwide for 36 cancers in 185 countries. CA Cancer J. Clin..

[2] Sawada N., Murata M., Kikuchi K., Osanai M., Tobioka H., Kojima T., Chiba H. (2003). Tight junctions and human diseases. Med. Electron. Microsc..

[3] Gonzalez-Mariscal L., Tapia R., Chamorro D. (2008). Crosstalk of tight junction components with signaling pathways. Biochim. Biophys. Acta.

[4] Tsukita S., Yamazaki Y., Katsuno T., Tamura A., Tsukita S. (2008). Tight junction-based epithelial microenvironment and cell proliferation. Oncogene.

[5] Mineta K., Yamamoto Y., Yamazaki Y., Tanaka H., Tada Y., Saito K., Tamura A., Igarashi M., Endo T., Takeuchi K. (2011). Predicted expansion of the claudin multigene family. FEBS Lett..

[6] Ouban A., Ahmed A.A. (2010). Claudins in human cancer: A review. Histol. Histopathol..

[7] Singh A.B., Sharma A., Dhawan P. (2010). Claudin family of proteins and cancer: An overview. J. Oncol..

[8] Valle B.L., Morin P.J. (2010). Claudins in Cancer Biology. Curr. Top Membr..

[9] Günzel D., Fromm M. (2012). Claudins and other tight junction proteins. Compr. Physiol..

[10] Brandner J.M., Zorn-Kruppa M., Yoshida T., Moll I., Beck L.A., De Benedetto A. (2015). Epidermal tight junctions in health and disease. Tissue Barriers.

[11] Hintsala H.R., Siponen M., Haapasaari K.M., Karihtala P., Soini Y. (2013). Claudins 1, 2, 3, 4, 5 and 7 in solar keratosis and squamocellular carcinoma of the skin. Int. J. Clin. Exp. Patho..

[12] Bello I.O., Vilen S.T., Niinimaa A., Kantola S., Soini Y., Salo T. (2008). Expression of claudins 1, 4, 5, and 7 and occludin, and relationship with prognosis in squamous cell carcinoma of the tongue. Hum. Pathol..

[13] dos Reis P.P., Bharadwaj R.R., Machado J., MacMillan C., Pintilie M., Sukhai M.A., Perez-Ordonez B., Gullane P., Irish J., Kamel-Reid S. (2008). Claudin 1 Overexpression Increases Invasion and Is Associated With Aggressive Histological Features in Oral Squamous Cell Carcinoma. Cancer Interdiscip. Int. J. Am. Cancer Soc..

[14] Lourenço S.V., Coutinho-Camillo C.M., Buim M.E.C., De Carvalho A.C., Lessa R.C., Pereira C.M., Vettore A.L., Carvalho A.L., Fregnani J.H., Kowalski L.P. (2010). Claudin-7 down-regulation is an important feature in oral squamous cell carcinoma. Histopathology..

[15] Melchers L., de Bruin L.B., Schnell U., Slagter-Menkema L., Mastik M., de Bock G., van Dijk B., Giepmans B., van der Laan B., van der Wal J. (2013). Lack of claudin-7 is a strong predictor of regional recurrence in oral and oropharyngeal squamous cell carcinoma. Oral Oncol..

[16] Nelhűbel G.A., Károly B., Szabó B., Lotz G., Kiss A., Tóvári J., Kenessey I. (2014). The prognostic role of claudins in head and neck squamous cell carcinomas. Pathol. Oncol. Res..

[17] Ouban A., Ahmed A. (2015). Analysis of the distribution and expression of claudin-1 tight junction protein in the oral cavity. Appl. Immunohistochem. Mol. Morphol..

[18] Al Moustafa A.-E., Alaoui-Jamali M.A., Batist G., Hernandez-Perez M., Serruya C., Alpert L., Black M.J., Sladek R., Foulkes W.D. (2002). Identification of genes associated with head and neck carcinogenesis by cDNA microarray comparison between matched primary normal epithelial and squamous carcinoma cells. Oncogene.

[19] Zhao X.Y., Sun S.Y., Zeng X.Q., Cui L. (2018). Expression profiles analysis identifies a novel three-mRNA signature to predict overall survival in oral squamous cell carcinoma. Am. J. Cancer Res..

[20] Lourenco S.V., Coutinho-Camillo C.M., Buim M.E., Pereira C.M., Carvalho A.L., Kowalski L.P., Soares F.A. (2010). Oral squamous cell carcinoma: Status of tight junction claudins in the different histopathological patterns and relationship with clinical parameters. A tissue-microarray-based study of 136 cases. J. Clin. Pathol..

[21] Babkair H., Yamazaki M., Uddin S., Maruyama S., Abé T., Essa A., Sumita Y., Ahsan S., Swelam W., Cheng J. (2016). Aberrant expression of the tight junction molecules claudin-1 and zonula occludens-1 mediates cell growth and invasion in oral squamous cell carcinoma. Hum. Pathol..

[22] Ouban A., Hamdan H., Hakam A., Ahmed A.A. (2012). Claudin-1 expression in squamous cell carcinomas of different organs: Comparative study of cancerous tissues and normal controls. Int. J. Surg. Pathol..

[23] Sappayatosok K., Phattarataratip E. (2015). Overexpression of Claudin-1 is Associated with Advanced Clinical Stage and Invasive Pathologic Characteristics of Oral Squamous Cell Carcinoma. Head Neck Pathol..

[24] Upadhaya P., Barhoi D., Giri A., Bhattacharjee A., Giri S. (2019). Joint detection of claudin-1 and junctional adhesion molecule-A as a therapeutic target in oral epithelial dysplasia and oral squamous cell carcinoma. J. Cell Biochem..

[25] Ivina A.A., Babichenko I.I., Rabinovich O.F., Togonidze A.A. (2014). Ki-67 and claudin-1 expression in hyperplasia, oral squamous inthraepithelial neoplasia and oral squamous cell carcinoma. Stomatologiia.

[26] Morita K., Tsukita S., Miyachi Y. (2004). Tight junction-associated proteins (occludin, ZO-1, claudin-1, claudin-4) in squamous cell carcinoma and Bowen’s disease. Br. J. Dermatol..

[27] Singh P., Toom S., Huang Y.W. (2017). Anti-claudin 18.2 antibody as new targeted therapy for advanced gastric cancer. J. Hematol. Oncol..

[28] Kimbung S., Kovács A., Bendahl P.-O., Malmström P., Fernö M., Hatschek T., Hedenfalk I. (2014). Claudin-2 is an independent negative prognostic factor in breast cancer and specifically predicts early liver recurrences. Mol. Oncol..

[29] Kinugasa T., Huo Q., Higashi D., Shibaguchi H., Kuroki M., Tanaka T., Futami K., Yamashita Y., Hachimine K., Maekawa S. (2007). Selective up-regulation of claudin-1 and claudin-2 in colorectal cancer. Anticancer Res..

[30] Wang Y.B., Shi Q., Li G., Zheng J.H., Lin J., Qiu W. (2019). MicroRNA-488 inhibits progression of colorectal cancer via inhibition of the mitogen-activated protein kinase pathway by targeting claudin-2. Am. J. Physiol. Cell Physiol..

[31] Okada T., Konno T., Kohno T., Shimada H., Saito K., Satohisa S., Saito T., Kojima T. (2020). Possibility of Targeting Claudin-2 in Therapy for Human Endometrioid Endometrial Carcinoma. Reprod. Sci..

[32] Moldvay J., Jackel M., Paska C., Soltesz I., Schaff Z., Kiss A. (2007). Distinct claudin expression profile in histologic subtypes of lung cancer. Lung Cancer.

[33] Venugopal S., Anwer S., Szaszi K. (2019). Claudin-2: Roles beyond Permeability Functions. Int. J. Mol. Sci..

[34] Dias M.C., Coisne C., Lazarevic I., Baden P., Hata M., Iwamoto N., Francisco D.M., Vanlandewijck M., He L., Stroka D. (2019). Claudin-3-deficient C57BL/6J mice display intact brain barriers. Sci. Rep..

[35] de Vicente J.C., Fernandez-Valle A., Vivanco-Allende B., Santamarta T.R., Lequerica-Fernandez P., Hernandez-Vallejo G., Allonca-Campa E. (2015). The Prognostic Role of Claudins-1 and-4 in Oral Squamous Cell Carcinoma. Anticancer Res..

[36] Bouchagier K.A., Assimakopoulos S.F., Karavias D.D., Maroulis I., Tzelepi V., Kalofonos H., Karavias D.D., Kardamakis D., Scopa C.D., Tsamandas A.C. (2014). Expression of Claudins-1,-4,-5,-7 and Occludin in Hepatocellular Carcinoma and their Relation with Classic Clinicopathological Features and Patients’ Survival. In Vivo.

[37] Sung C.O., Han S.Y., Kim S.H. (2011). Low Expression of Claudin-4 is Associated with Poor Prognosis in Esophageal Squamous Cell Carcinoma. Ann. Surg. Oncol..

[38] Phattarataratip E., Sappayatosok K. (2016). Expression of claudin-5, claudin-7 and occludin in oral squamous cell carcinoma and their clinico-pathological significance. J. Clin. Exp. Dent..

[39] Yoshizawa K., Nozaki S., Kato A., Hirai M., Yanase M., Yoshimoto T., Kimura I., Sugiura S., Okamune A., Kitahara H. (2013). Loss of claudin-7 is a negative prognostic factor for invasion and metastasis in oral squamous cell carcinoma. Oncol. Rep..

[40] Li X., Yang W.D. (2022). IRF2-induced Claudin-7 suppresses cell proliferation, invasion and migration of oral squamous cell carcinoma. Exp. Ther. Med..

[41] Cristaldi M., Mauceri R., Di Fede O., Giuliana G., Campisi G., Panzarella V. (2019). Salivary Biomarkers for Oral Squamous Cell Carcinoma Diagnosis and Follow-Up: Current Status and Perspectives. Front. Physiol..

