# Supplementary material for: Clinical Significance of Claudin Expression in Oral Squamous Cell Carcinoma
Source: Int J Mol Sci. 2022 Sep 23;23(19):11234. doi: 10.3390/ijms231911234 (PMC9569574; doi:10.3390/ijms231911234)

Figure S1: Analysis of claudin-3 expression in 60 controls and respective tumors using Western blots. Western blot analysis of claudin-3 in 60 controls and associated tumors, one band was always detected at approx. 55 kDa and shown here with loading control β-actin (42 kDa) below. Further analysis using pulldown assay and mass spectroscopy showed that this band is not claudin-3. Patients 19, 49, 55 are shown for the sake of completeness, but these are the oropharynx carcinomas that were excluded in this study.

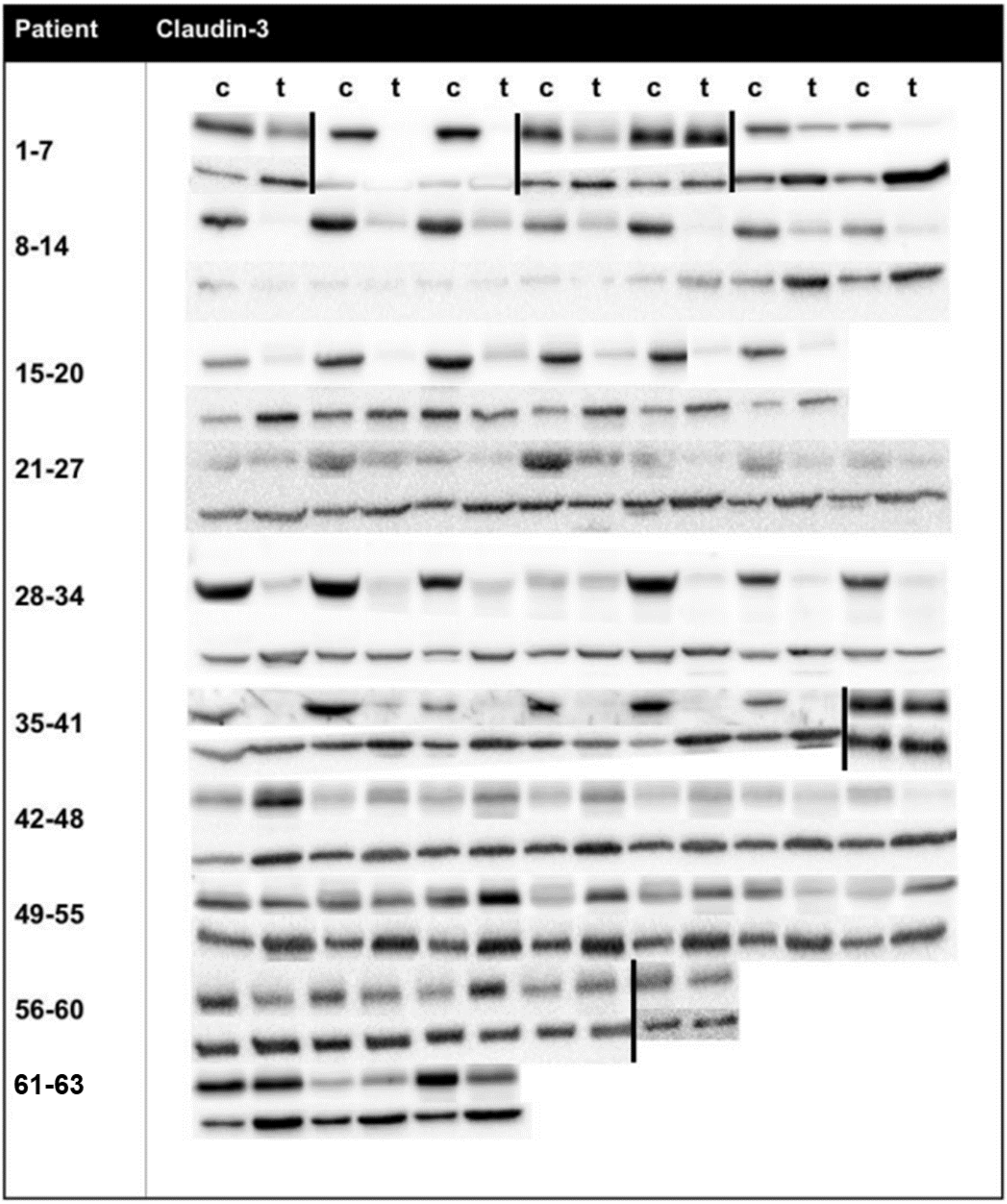

Supplement: Supplementary file 1 [file ijms-23-11234-s001.zip › Figure S1.pdf]
