# Supplementary material for: Clinical Significance of Claudin Expression in Oral Squamous Cell Carcinoma
Source: Int J Mol Sci. 2022 Sep 23;23(19):11234. doi: 10.3390/ijms231911234 (PMC9569574; doi:10.3390/ijms231911234)

Figure S2: Claudin expression differences in various parts of the oral cavity. Claudin expression differences between control and tumor tissue analyzed in Western Blot studies grouped in various areas of the oral cavity. The claudin expression in tumor tissue is higher (positive values) or lower (negative values) as in control tissue (=0). The mean difference of intensity between tumor and control after normalisation with β-actin is shown, error bars: 95% confidence interval.

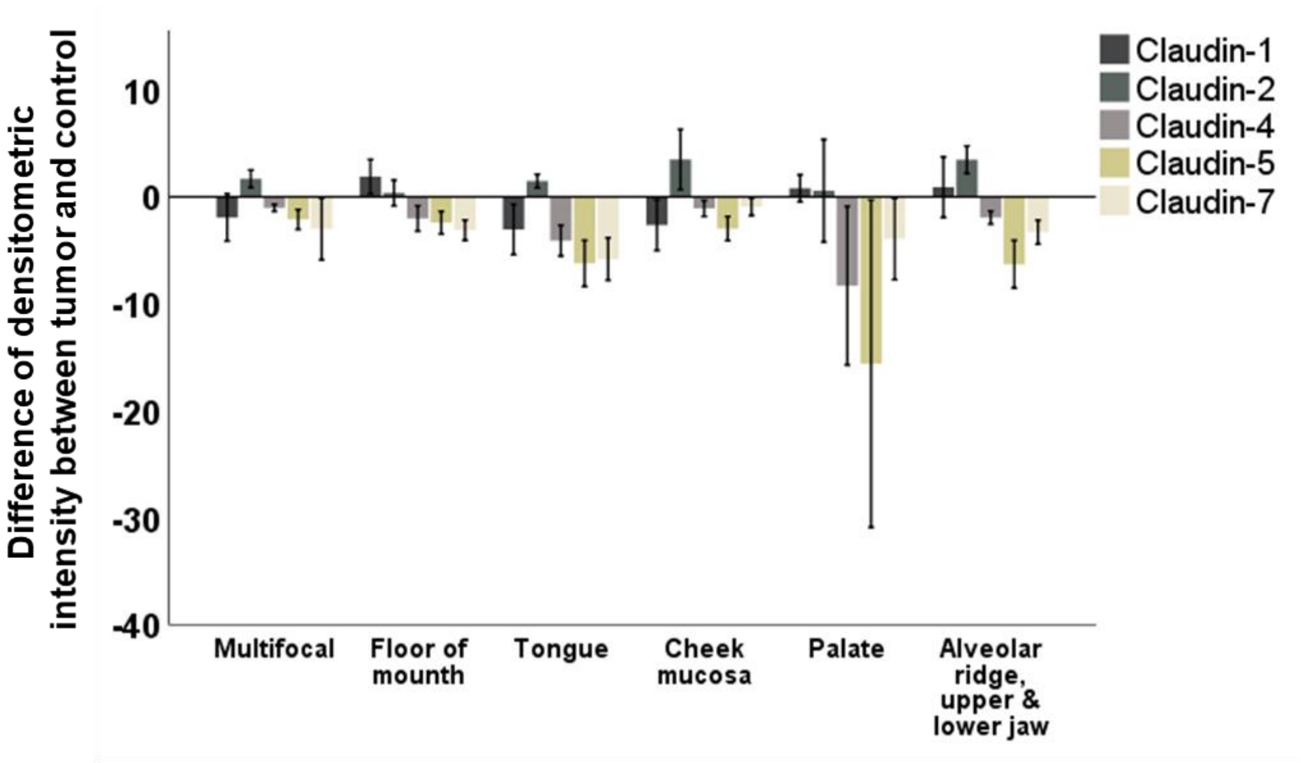

Supplement: Supplementary file 1 [file ijms-23-11234-s001.zip › Figure S2.pdf]
