# Supplementary material for: Clinical Significance of Claudin Expression in Oral Squamous Cell Carcinoma
Source: Int J Mol Sci. 2022 Sep 23;23(19):11234. doi: 10.3390/ijms231911234 (PMC9569574; doi:10.3390/ijms231911234)
Supplement: Supplementary file 1 [file ijms-23-11234-s001.zip › Figure S3.pdf]

Figure S3: Analysis of claudin-1, -2, -4, -5, -7 expression in 60 controls and respective tumors using Western blots.

Western blot analysis of claudin-1 (23 kDa), claudin-2 (24 kDa), claudin-4 (22 kDa), claudin-5 (23 kDa, in the case of double bands the lower band) and claudin-7 (22 kDa) with loading control β-actin (42 kDa) directly below the claudin bands. Patients 19, 49, 55 are shown for completeness, these are oropharyngeal carcinomas, which were excluded from this study.

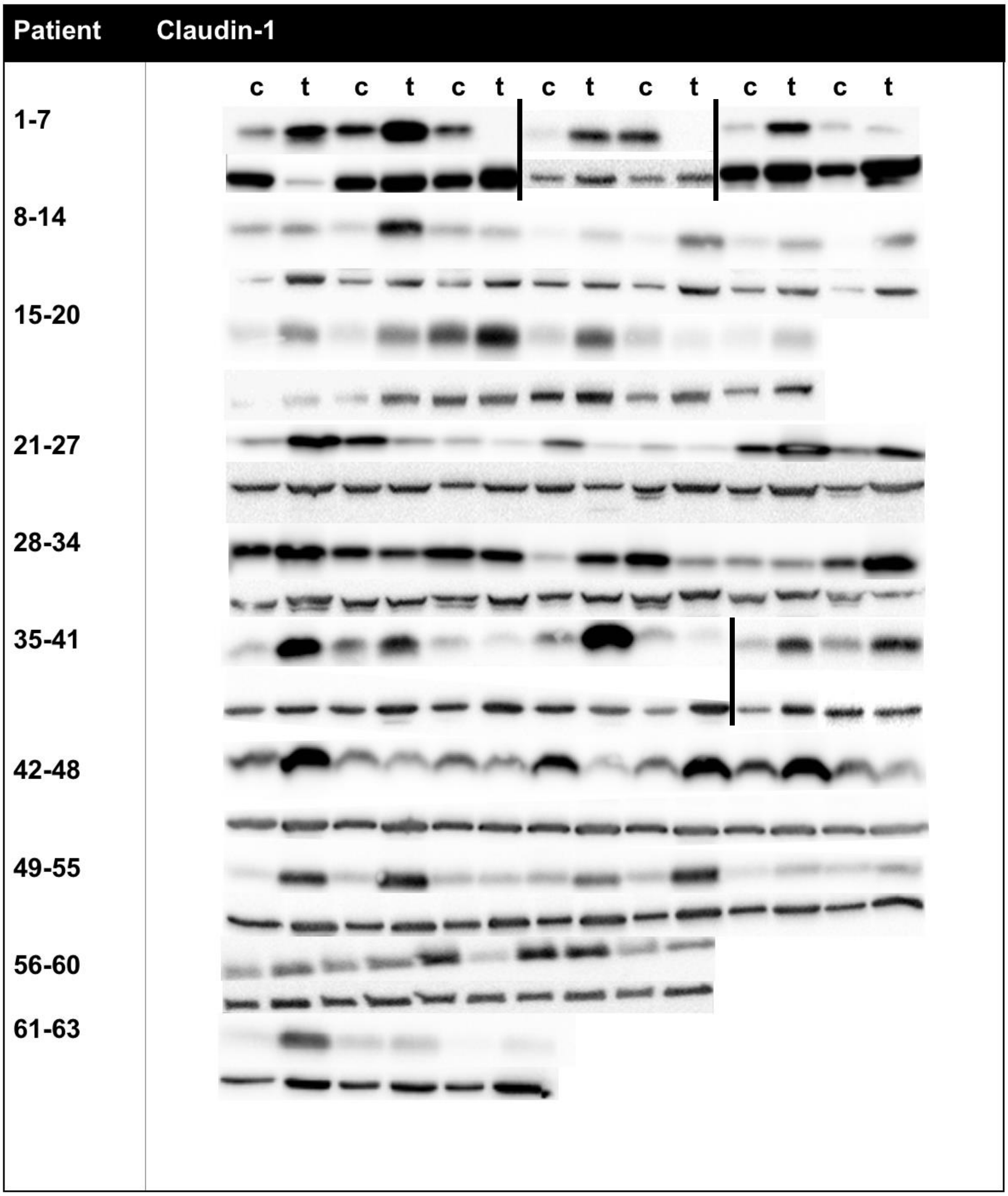

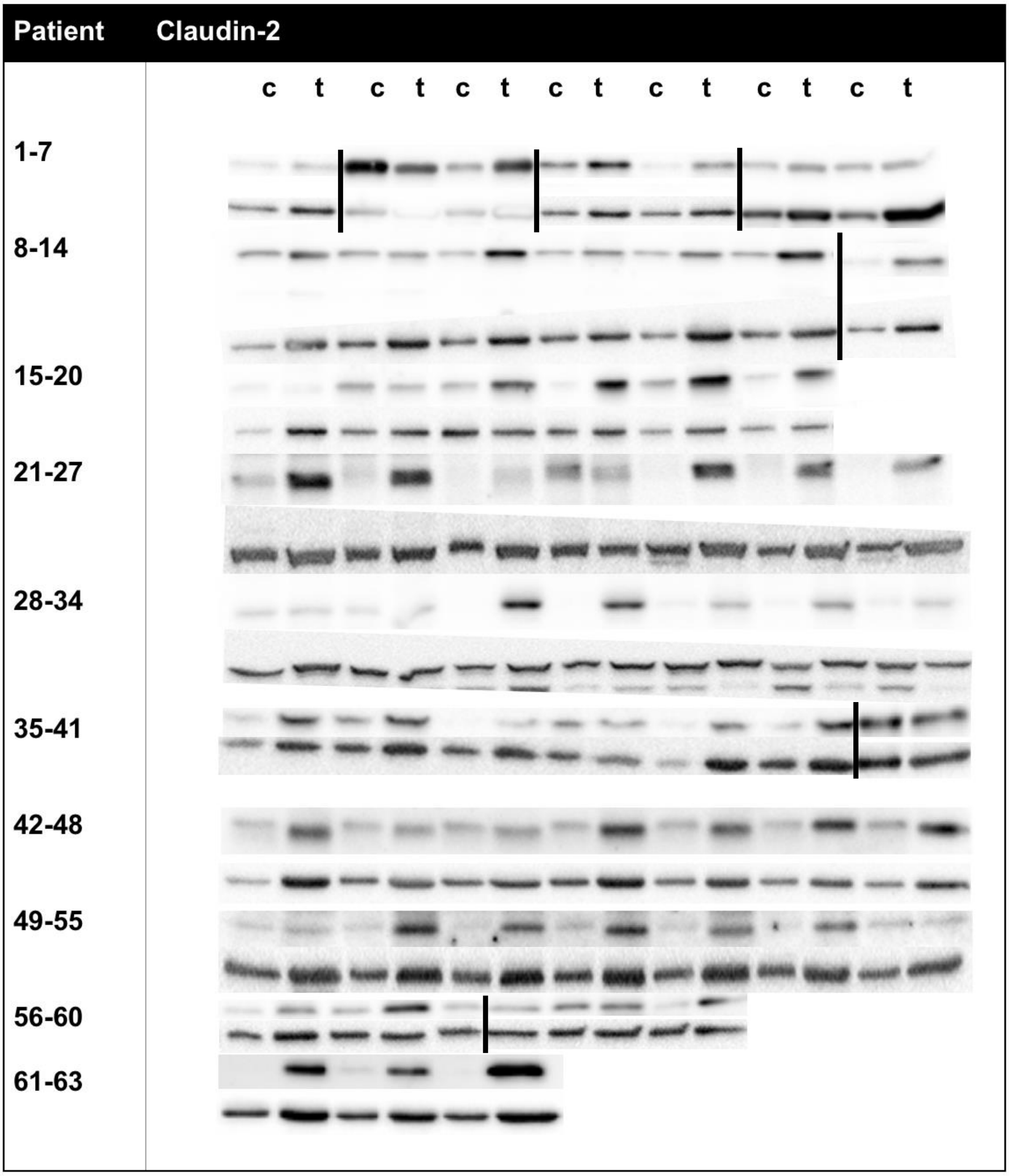
Patient
Claudin-2
c t c t c t c t c t c t c t
1-7
8-14
15-20
21-27
28-34
35-41
42-48
49-55
56-60
61-63

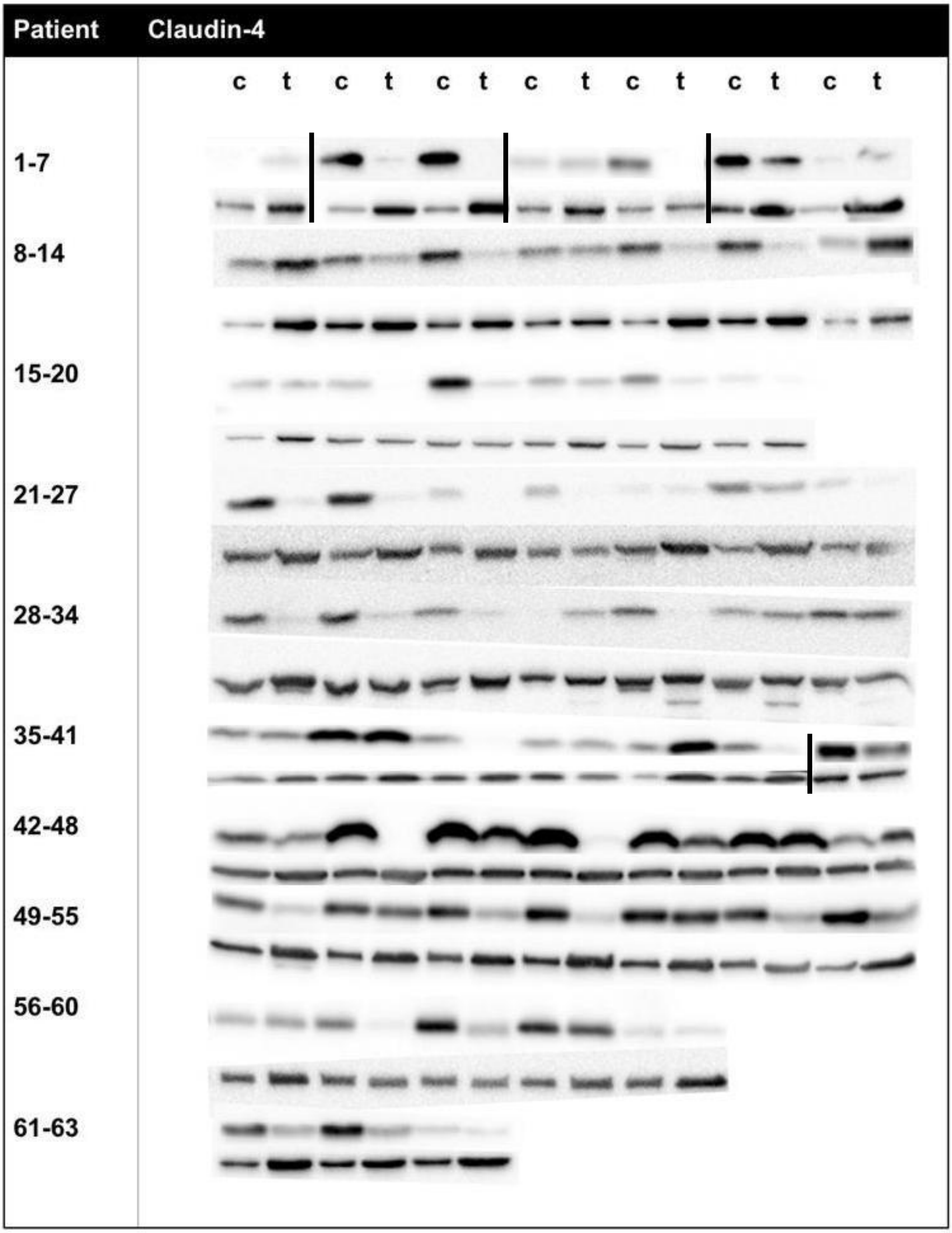

Patient
Claudin-4
c t c t c t c t c t c t c t
1-7
8-14
15-20
21-27
28-34
35-41
42-48
49-55
56-60
61-63

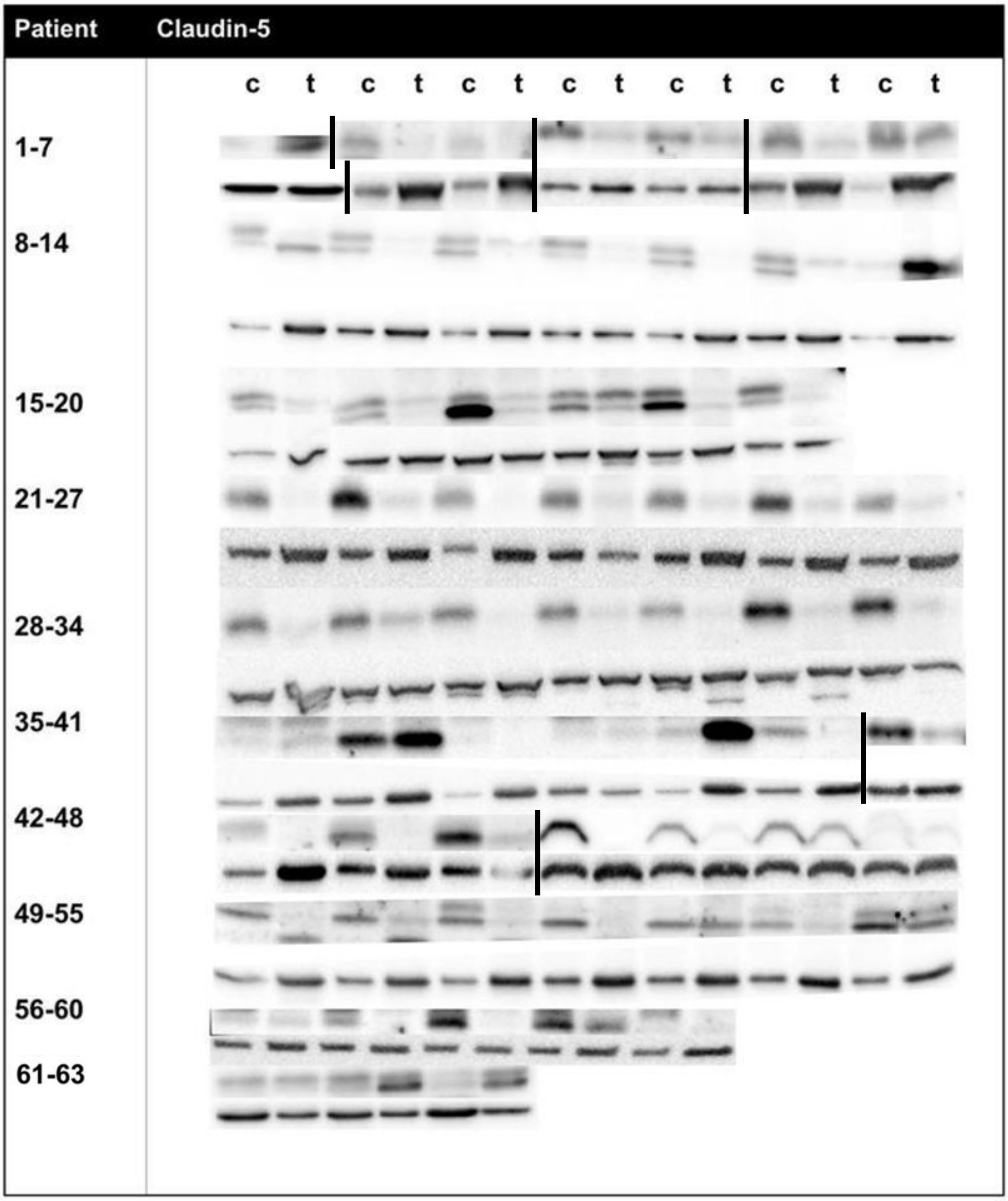
Patient
Claudin-5
c t c t c t c t c t c t c t
1-7
8-14
15-20
21-27
28-34
35-41
42-48
49-55
56-60
61-63

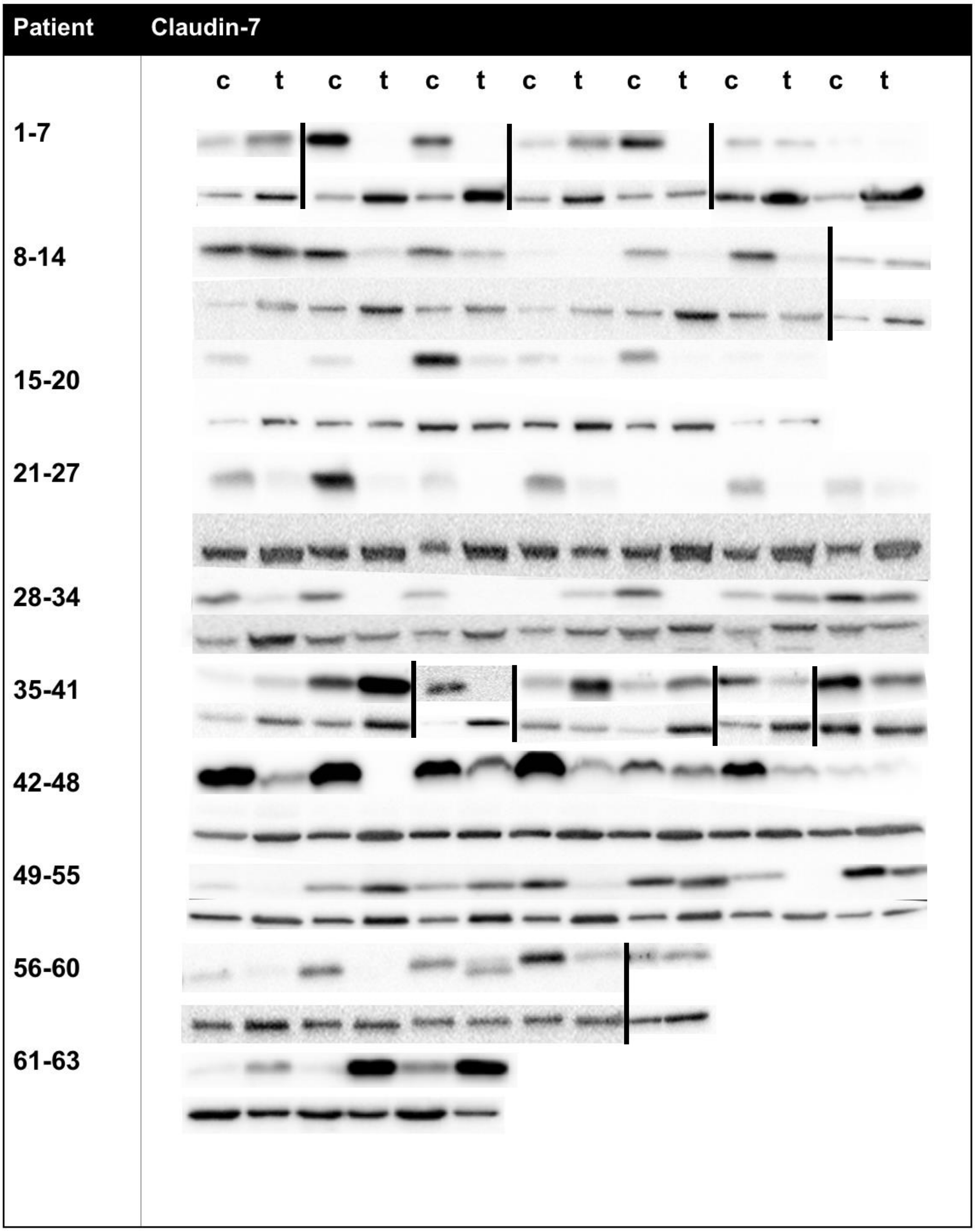
Patient
Claudin-7
c t c t c t c t c t c t c t
1-7
8-14
15-20
21-27
28-34
35-41
42-48
49-55
56-60
61-63
