# Supplementary material for: Clinical Significance of Claudin Expression in Oral Squamous Cell Carcinoma
Source: Int J Mol Sci. 2022 Sep 23;23(19):11234. doi: 10.3390/ijms231911234 (PMC9569574; doi:10.3390/ijms231911234)
Supplement: Supplementary file 1 [file ijms-23-11234-s001.zip › Table S1.pdf]

Table S1. Classification of lymph node status according to UICC 2017 (8th edition)

Lymph note status	Number	%
N0	27	45
N1	6	10
N2 a-c	5	8.3
N3 a-b	9	15
Not specified	13	21.7
Total	60	100
