# Supplementary material for: Clinical Significance of Claudin Expression in Oral Squamous Cell Carcinoma
Source: Int J Mol Sci. 2022 Sep 23;23(19):11234. doi: 10.3390/ijms231911234 (PMC9569574; doi:10.3390/ijms231911234)
Supplement: Supplementary file 1 [file ijms-23-11234-s001.zip › Table S2.pdf]

Table S2. Comparison of lymph node status, classified according to UICC 2009 and UICC 2017, and claudin expression in the OSCC.

Clinicopathological features	Number	Claudin-1	Claudin-2	Claudin-4	Claudin-5	Claudin-7
**Lymph node status** (N0 - N2a,b,c) TNM classification 2009	54	r = 0.150 p = 0.279	r = -0.018 p = 0.898	r = -0.014 p = 0.920	r = 0.010 p = 0.940	r = -0.387 **p = 0.004****
**Lymph node status** (N0 – N3a,b) TNM classification 2017	47	r = 0.036 p = 0.811	r = -0.033 p = 0.829	r = -0.039 p = 0.795	r = 0.099 p = 0.515	r = -0.404 **p = 0.005****

Calculation with the Spearman-Rho or Mann-Whitney-U test with a significant result at ** $p < 0.01$. (r = rank correlation coefficient).
