# Supplementary material for: Clinical Significance of Claudin Expression in Oral Squamous Cell Carcinoma
Source: Int J Mol Sci. 2022 Sep 23;23(19):11234. doi: 10.3390/ijms231911234 (PMC9569574; doi:10.3390/ijms231911234)
Supplement: Supplementary file 1 [file ijms-23-11234-s001.zip › Table S3.pdf]

Table S3: Group classification for Kaplan-Meier-survival analysis

Expression difference	Claudin-2	Claudin-4
**Median**	0.5166	-1.2990
**Percentile**		
33.3	0.0791	-1.7837
66.7	1.5743	-0.4159
