# Supplementary material for: Clinical Significance of Claudin Expression in Oral Squamous Cell Carcinoma
Source: Int J Mol Sci. 2022 Sep 23;23(19):11234. doi: 10.3390/ijms231911234 (PMC9569574; doi:10.3390/ijms231911234)
Supplement: Supplementary file 1 [file ijms-23-11234-s001.zip › Table S4.pdf]

Table S4. Cox-multi regression analysis after reclassification of lymph node status

**Clinicopathological features**	**B**	**Significance**	**Exp(B)**
Claudin-2 difference	0.286	**0.006***	1.331
Age at diagnosis	0.018	0.618	1.019
T stage	-0.275	0.526	0.759
Lymph node status	0.709	**0.006***	2.031
Histological differentiation	-0.611	0.473	0.543

Claudin-2 expression difference in healthy and tumor tissue, lymph node status function as independent variable in relapse-free survival. Significant result at * $p < 0.05$. (B - regression coefficient, Exp(B) Odds ratio).
