# Supplementary material for: Clinical Significance of Claudin Expression in Oral Squamous Cell Carcinoma
Source: Int J Mol Sci. 2022 Sep 23;23(19):11234. doi: 10.3390/ijms231911234 (PMC9569574; doi:10.3390/ijms231911234)
Supplement: Supplementary file 1 [file ijms-23-11234-s001.zip › Table S5.pdf]

Table S5. Evaluation of cases for lymph node classification according to UICC 2017

Available cases for analysis es	Number	%	Cases not used for analysis	Number	%
**Recurrence-free time[a]**	10	16.7	**Cases with missing values**	15	25
**Censored**	26	43.3	**Censored before earliest relapse**	9	15
**In total**	36	60	**In total**	24	40

[a] Dependent variable: Recurrence-free time
